# Hybrid Basketball Game Outcome Prediction Model by Integrating Data Mining Methods for the National Basketball Association

**DOI:** 10.3390/e23040477

**Published:** 2021-04-17

**Authors:** Wei-Jen Chen, Mao-Jhen Jhou, Tian-Shyug Lee, Chi-Jie Lu

**Affiliations:** 1 Graduate Institute of Business Administration, Fu Jen Catholic University, New Taipei City 242062, Taiwan; justice1207@gmail.com (W.-J.C.); aaa73160@gmail.com (M.-J.J.); 036665@mail.fju.edu.tw (T.-S.L.); 2 Artificial Intelligence Development Center, Fu Jen Catholic University, New Taipei City 242062, Taiwan; 3 Department of Information Management, Fu Jen Catholic University, New Taipei City 242062, Taiwan

**Keywords:** sports outcomes prediction, basketball game, game score prediction, data mining, XGBoost, National Basketball Association

## Abstract

The sports market has grown rapidly over the last several decades. Sports outcomes prediction is an attractive sports analytic challenge as it provides useful information for operations in the sports market. In this study, a hybrid basketball game outcomes prediction scheme is developed for predicting the final score of the National Basketball Association (NBA) games by integrating five data mining techniques, including extreme learning machine, multivariate adaptive regression splines, k-nearest neighbors, eXtreme gradient boosting (XGBoost), and stochastic gradient boosting. Designed features are generated by merging different game-lags information from fundamental basketball statistics and used in the proposed scheme. This study collected data from all the games of the NBA 2018–2019 seasons. There are 30 teams in the NBA and each team play 82 games per season. A total of 2460 NBA game data points were collected. Empirical results illustrated that the proposed hybrid basketball game prediction scheme achieves high prediction performance and identifies suitable game-lag information and relevant game features (statistics). Our findings suggested that a two-stage XGBoost model using four pieces of game-lags information achieves the best prediction performance among all competing models. The six designed features, including averaged defensive rebounds, averaged two-point field goal percentage, averaged free throw percentage, averaged offensive rebounds, averaged assists, and averaged three-point field goal attempts, from four game-lags have a greater effect on the prediction of final scores of NBA games than other game-lags. The findings of this study provide relevant insights and guidance for other team or individual sports outcomes prediction research.

## 1. Introduction

The sports market has grown rapidly over the last several decades with the development of technology, broadcasting, press, and social media. The prediction of sports outcomes is crucial in many sports markets, such as sports betting, club management and operations, and broadcast management, since precise sports outcomes prediction provides accurate betting reference, management and operations information, and increased viewer interests. For example, in sports lotteries, there will be more interest in predicting scores as the major bets focus on scores, such as spread points, handicaps, correct scores, or total over/under goals. Therefore, developing an effective sports outcomes prediction model that can achieve accurate and robust prediction results is one of the important and attractive challenges of sports analytics [1].

Sports outcomes prediction has attracted attention in different sports [1,2,3,4,5,6]. However, most of the existing studies in this regard are focused on basketball, in particular, on the National Basketball Association (NBA) games, since the NBA is the most popular basketball league in the world [4,6,7,8,9,10,11,12,13,14,15]. Most existing research related to basketball games is aimed at predicting the win/lose (W/L) outcomes of a game [3,4,6,8,9,10,12,15]. Only a few studies have focused on the prediction of basketball game scores [11,12,13,14,15].

The W/L prediction provides only binary classification results; whereas, score prediction provides the intensity of a basketball game, i.e., more detailed information for stakeholders and relative application, such as sports betting, club management and operations, and broadcast management. In this study, we integrate data mining methods to propose a hybrid NBA game score prediction model.

Data mining, the process of automatically exploring potentially useful information in a large dataset, has been successfully used to construct effective forecast models for different applications in various fields [16]. There are few articles utilizing data mining methods for NBA game outcomes prediction [8,9,10,12,15]. Only the authors of [13] implemented data mining methods on NBA game score prediction. The five well-known data mining methods, including multivariate adaptive regression splines (MARS), k-nearest neighbors (KNN), extreme learning machine (ELM), eXtreme gradient boosting (XGBoost), and stochastic gradient boosting (SGB), are used in this study for building an NBA game score prediction model, as they have been widely used in various applications such as public health [17,18], finance [19,20] and civil engineering [21,22]. Moreover, the five methods are also successfully applied in the sports outcomes prediction research [6,23,24,25,26].

Regarding the five data mining methods, MARS, a powerful nonparametric regression method, can identify model relationships that are nearly additive or involve interactions with fewer variables [27]. KNN is a lazy algorithm that solves classification and regression problems by assigning weight to the contributions of the neighbors, where nearer neighbors contribute more than others [28]. ELM is a simple and efficient single-hidden-layer feedforward neural network [29]. XGBoost is a supervised learning algorithm based on a scalable end-to-end gradient tree boosting system [30]. SGB is a gradient-boosting-based algorithm that adds bagging and randomness into the tree building procedure, using full-dataset subsampling [31].

In the proposed hybrid basketball game score prediction model, we collected the most commonly used statistics of an NBA game as variables; then, relevant features derived from different game-lag information of a variable were generated. Since feature selection is useful for reducing the complexity of computation, the three embedded data mining feature selection techniques, including MARS, SGB, and XGBoost, are used to select relevant features for our hybrid model. After integrating and selecting the most important features, which are used as predictors, the MARS, KNN, ELM, SGB, and XGBoost, were implemented to generate the final models for predicting the final score of the NBA game. Finally, by comparing the prediction performances of the developed models under different game-lags and several selected important features, the best prediction model was identified, and the corresponding game-lag information and features used are considered the most suitable game-lag information and features for the NBA game score prediction.

The rest of this paper is organized as follows: Section 2 present the related literature review. Section 3 gives a brief introduction to the five algorithms used in this paper. Section 4 demonstrates the details of the proposed scheme. Results are presented in Section 5, followed by the Conclusions section.

## 2. Literature Review

Regarding the sports-market-related research, the research domain appears dynamic. Some studies paid attention to NBA policy such as players’ labor rights [32,33] and the decision-making process of the NBA draft [34,35]. Some research aims to determine the factors that affect the outcomes of sports games such as home advantage [36,37] and individual players’ performance [38,39].

There are many different prediction techniques have been applied in different areas, including civil engineering [40], industrial engineering [41,42], healthcare [43,44], safety [45] and data mining [46,47]. Sports outcomes prediction has attracted attention in different sports [1,2,3,4,5,6]. For example, the author of [7] built a simple, weighted and penalized regression model using the match-up, date and final score records to predict baseball, basketball, American football and hockey outcomes. However, most of the existing studies in this regard are focused on basketball, in particular, on the National Basketball Association (NBA) games, since NBA is the most popular basketball league in the world [4,6,8,9,10,11,12,13,14,15].

Part of sports outcomes prediction aims to predict the winners and losers of specific NBA games. The author of [8] proposed a fusion model using multiple neural network methods to predict the outcomes of NBA games. In [9], the author generated a scheme based on the maximum entropy principle and k-means clustering to predict the winner of NBA playoff games. The author of [10] presented a model based on the support vector machine, merging the decision tree and correlation-based feature selection algorithm to predict the outcomes of the NBA games. The author of [12] proposed a framework that used historical data of NBA finals games to build up a machine learning (ML) method model to predict the outcomes of NBA games. The author of [15] presented a model that used both basketball statistics on players and teams. They used multivariate logistic regression analysis to simulate players’ rotation and use these simulation results, i.e., the historical data of players and teams, as input data for predicting the outcomes of NBA games.

Some studies focus on the prediction of basketball game scores. The author of [11] established a regression model based on bivariate normal to investigate the relationship between an NBA team’s score and the team’s performance statistics. The author of [13] built a model based on regression tree, linear regression and support vector regression to predict the final score of the Golden State Warriors (an NBA team) in the 2017–2018 season. The author of [14] proposed a model based on the gamma process to predict the total points of NBA games, predicting the final total score of both teams.

## 3. Methods

### 3.1. MARS Algorithm

MARS, an adaptable algorithm used to discover the optimal transformations and interactions of variables, has been widely used to recognize model relationships that are additive or include interactions with fewer variables. It is a nonparametric statistical algorithm developed from the divide-and-conquer concept for segmenting training-data subsets into divided groups with their regression equations. The MARS model implements its nonlinearity using separate linear regression slopes in distinct intervals of the independent variable space.

MARS consists of a series of weighted sum of the basis functions (BFs), which are splines piecewise polynomial functions, and are demonstrated in the following equation [18,27]:

(1) $$f\left(x\right)=\alpha_{0}+\sum_{m}^{M}a_{m}B_{m}\left(x\right)$$

where $a_{0}$ and $a_{m}$ are constant coefficients that can be estimated using a least-squares method. M is the total number of BFs. $B_{m}\left(x\right)\;$ represents the BFs. The hinge functions, *max* (0, *x − k*) or *max* (0, *k − x*), with a knot defined at value *t*, are used in MARS modeling [48].

### 3.2. ELM Algorithm

ELM, a single-hidden-layer feedforward neural network that randomly determines the input weights and systematically computes the output weights of the network [28], has a faster modeling time than the conventional feedforward network learning algorithms. It reduces usual disadvantages found in gradient-based methods, such as stopping criteria, learning rate and epochs [18].

Input weights and hidden layer biases in the ELM algorithm can be randomly generated, and the determination of output weights is as simple as finding the least-square solution for a specific linear system. Therefore, a linear system achieves its minimum norm least-square solution by $\;\hat{A}=\tilde{H}$, where $\tilde{H}\;$represents the Moore–Penrose generalized inverse of the original matrix H. The minimum norm least-squares solution has the smallest norm among all other solutions [18,29].

### 3.3. XGBoost Algorithm

XGBoost, one of the widely used tree-based learning methods, is a supervised ML algorithm developed from a scalable end-to-end gradient tree boosting concept [30]. Boosting is an ensemble learning technique that develops many models concurrently, with each new model aiming to improve the disadvantage of the previous model. A weak learner (ML model) is developed to be maximally correlated with the negative gradient of the loss function related to the entire scheme for each iteration in gradient boosting [18,49].

XGBoost is an application of a generalized gradient boosting decision tree that is implemented by a new distributed tree searching method that reduces tree construction time. XGBoost moderates overfitting and provides support for arbitrary adaptable loss functions by regularization term [18,50].

### 3.4. KNN Algorithm

KNN, a supervised data mining and ML method, is useful for solving classification and regression problems. Both classification and regression methods design weights based on the influence of neighbors, where the nearest neighbors have more influence than the others. The fundamental of KNN is the metric distance value, for which various metrics have been developed; the most common metric is the Euclidean distance [28].

KNN finds a group of k objects in the training set that are closest to the test object and facilitates the assignment of a label based on the prevalence of a specific class in this neighborhood [16,51]. For model-based methods, which learn from training datasets and then predict test datasets with the trained models, the KNN method reduces the training stage and performs classification tasks by computing the distance between test data points and all training data points to obtain the nearest neighbors and then proceed with the KNN classification [52].

### 3.5. SGB Algorithm

In SGB, a hybrid method that comprises boosting and bagging techniques [53,54], data are selected by random sampling at each stage of the steepest gradient algorithm-based boosting procedure. Smaller trees are developed instead of developing a full classification tree at each stage of the boosting process [55]. Optimal data fractionation is calculated by referring to a consequential process, and the residual of each fraction is determined. The next step in finding a new fraction, which is expected to reduce the variance of the residual of the data from the tress sequence, is to fit the residual tree. The results are merged to reduce the sensitivity of these methods for target datasets [56]. SGB does not require pre-select or transform predictor variables and is resistant to outliers since the steepest gradient methods concentrate on points that are similar to their correct classification [57].

### 3.6. Model Implementation

In this study, all methods were implemented in R version 3.6.2 [58]. MARS was implemented by the *earth* package version 5.3.0 [59]. The default setting of this package was set. XGBoost was implemented by the *XGBoost* package version 1.3.2.1 [60]. To estimate the best parameter set for developing effective XGBoost models, the *caret* package version 6.0-84 was used for tuning the relevant hyperparameters [61]. SGB was constructed by the *gbm* package version 2.1.8 [62]. ELM was computed by the *elmNN* package version 1.0 [63]. The default activation function used in this package is radial bias. The *caret* package version 6.0-84 was also implemented to search for the best number of hidden neurons that can generate promising ELM models [61]. KNN was implemented by the *kknn* package version 1.3.1 [64].

## 4. Proposed Basketball Game Score Prediction Scheme

**Figure 1:**
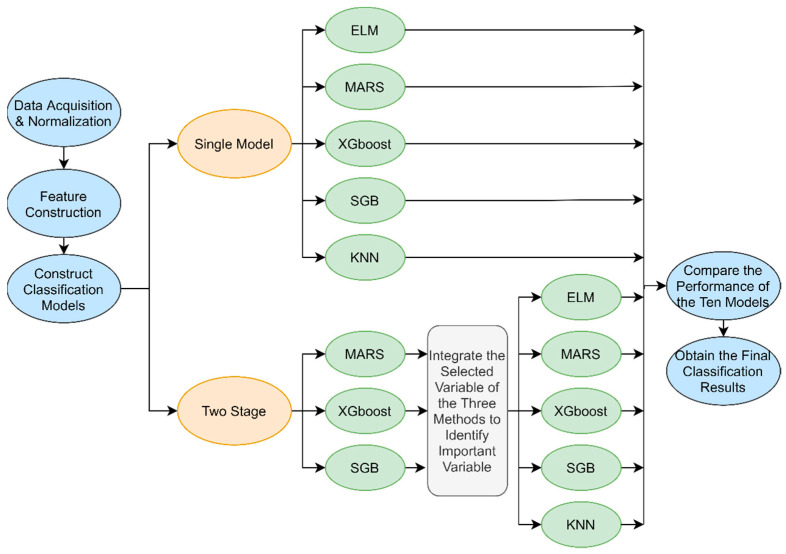
Flowchart of the proposed basketball game score prediction scheme.

In this study, the five data mining techniques described above were integrated to develop a novel scheme for predicting the final score of an NBA game. The flowchart of the proposed scheme is shown in Figure 1.

The first step of the proposed scheme was data acquisition and normalization. We collected data from the basketball-reference website (https://www.basketball-reference.com, accessed on 15 March 2021) [65] for every single NBA game in the 2018–2019 season. That NBA season comprised 1230 games, and each game is categorized into home/away team statistics. Each game generates two datasets, one from the home and another from the away team. Therefore, 2460 game scores were collected and used in our research.

A total of 14 variables were collected and used in this study. One is the final score of a team; the remaining 13 are the most commonly used statistics of a game, such as the team’s defensive performance and game-related offenses [4,6,8,9,10,11,12,13,14,15]. Table 1 shows variable definitions; variable $V_{i,t}\;$is the *i*-th variable at the *t*-th game and variable $Y_{t}$ is the final score at the *t*-th game, which is used as the target variable of this study. Since each team play 82 games in a season, variable $V_{i,t}\;$can be defined as 1≤i≤13, 1≤ t≤82.

Data normalization shall be implemented before data analysis since different variables have different scales. The min-max normalization method was used to convert a value v of each variable V to $v^{\prime }$ in the range [0, 1] by calculating using the following equation:

(2) $$V_{i,t}^{\prime }=\frac{V_{i,\;t}-minV_{i}}{maxV_{i}-minV_{i}}$$

where $maxV_{i}$ and *min*$V_{i}$ are the maximum and minimum values for the attribute $V_{i}$. Data normalization was performed to ensure that large input variable values do not influence smaller input values, thus reducing prediction errors.

The second step is the feature construction to generate input features for the data mining models based on the variables shown in Table 1. We define the game-lag of a game variable as “the *n*-th game before game *t*”. For example, the third game-lag of game 65 is game 62. In most related research, researchers used only the game-lag information of up to six games for model construction [8,9]. To consider more complete game-lag information, the game-lag information of 1–6 games is used in this study. However, a variable value in a single game may not be sufficient for evaluating a team’s performance. Therefore, we calculate the mean value of a variable within l game-lags to evaluate a team’s performance during a specific period. Variable ${\bar{V}}_{i,t}^{l}$ is the designed *i*-th predictor variable at the *t*-th game with l game-lags.

(3) $${\bar{V}}_{i,t}^{l}=\frac{\sum_{n=1}^{l\;}V_{i,t-n}^{\prime }}{l},\forall \;i,\;t,n,\;l\;\in \mathbb{N},$$

where n is the *n*-th game-lag, 1 ≤ i ≤ 13, 1≤ l≤ 6, 1 ≤ t ≤ 82, n ≤ l.

**Figure 2:**
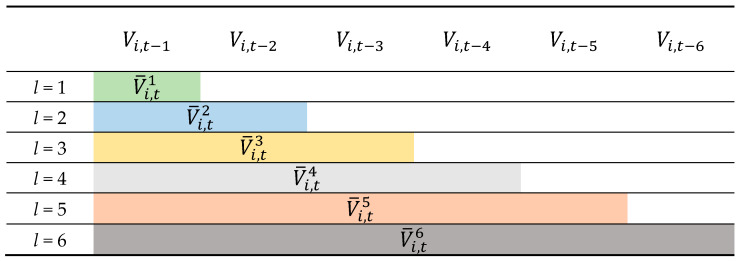
Example of the designed features for variable $V_{i,\;t}$ in different game-lags.

For instance, for the first variable (i = 1), if we want to design a feature considering three game-lags’ information (l = 3) for the game No. 10 (or 10-th game) (*t* = 10) of a team, the values of the first variable in the previous three games are averaged as the designed feature. That is$,\;{\bar{V}}_{1,10}^{3}=\frac{V_{1,9\;}+V_{1,8}+V_{1,7}}{3}$. Therefore, using the same concept, a variable in one game can be extended to six designed features under the consideration of one game-lag to six game-lags’ information. Figure 2 shows designed feature examples for variable $V_{i,\;t}$ in different game-lags.

This research aims to construct the prediction model using the designed features (${\bar{V}}_{i,t}^{l}$) to predict the final score of a game ($Y_{t}$), as expressed using Equation (4):

(4) $$Y_{t}=f\left({\bar{V}}_{i,t}^{l}\right),$$

where 1 ≤ i ≤ 13, 1 ≤ l ≤ 6, 7≤ t ≤ 82, ∀ i, t, l ∈ ℕ.

Note that all 13 designed features (1 ≤ i ≤ 13) were used with 1–6 game-lags’ information (1 ≤ l ≤ 6) for each $Y_{t}$. Since we use up to six games’ information as our game-lag information, the first six games of the season are skipped (7 ≤ t≤ 82).

In the third step, we construct predictive models for predicting final scores of the NBA games considering different game-lags. The predictive models were constructed using two types of modeling processes. One, a single modeling process, and the other, a two-stage modeling process. In the single modeling process, all 13 designed features were directly used as predictors for developing ELM, MARS, XGBoost, SGB, and KNN as five single-predictive models. These were termed single ELM (S-ELM), single MARS (S-MARS), single XGBoost (S-XGBoost), single SGB (S-SGB) and single KNN (S-KNN) models.

Developing a two-stage model began with the implementation of a feature selection method, as some important basketball variables have a greater influence on predicting the outcomes of basketball games. This study used an embedded feature selection method with the implementation of MARS, XGBoost, and SGB since these methods are equipped with feature selection functions. The three algorithms generate their best subsets of features. This study uses ensemble techniques to merge the selected features subsets of the three algorithms in order to provide stable and effective feature selection result. Ensemble technique is a paradigm, where several intermediate selected features are generated and combined using combination rules to obtain a simple selection result. It can be used to avoid unstable selection results and improve the performance of feature selection [66].

For example, Table 2 illustrates the feature importance ranking generated by MARS, XGBoost and SGB algorithms under game-lag = 4 (l = 4). Note that a feature with a rank of 1 is considered the most important, while one with a rank of 13 is considered less important than other features. The average ranking is obtained by calculating each feature by its ranking in the MARS, XGBoost and SGB. Table 2 shows the average rank of each feature. It can be observed that ${\bar{V}}_{8,t}^{4}$, with an average rank of 1.67, is the most important feature, followed by ${\bar{V}}_{2,t}^{4}$ with value 2.00 and ${\bar{V}}_{6,t}^{4}$ with value 3.67.

Table 2 illustrates the importance ranking of each designed feature. To reduce the number of less important features selected, this study uses the significant predictive feature selection rule proposed by the author of [67]. Their method selects important features based on the total number of features. If the total number of features is between 10 and 75, researchers can select 40% of the features as relevant from the overall features. Therefore, since there are 13 designed features in this paper, we select 6 designed features as relevant features.

According to the ranking results in Table 2, under game-lag = 4, ${\bar{V}}_{8,t}^{4}$, ${\bar{V}}_{2,t}^{4}$, ${\bar{V}}_{6,t}^{4},$ ${\bar{V}}_{7,t}^{4},$ ${\bar{V}}_{9,t}^{4}$ and ${\bar{V}}_{3,t}^{4}$ are selected as the relevant features. These six relevant features served as the input variables for the ELM, MARS, XGBoost, SGB and KNN methods for predicting the final score of an NBA game. The five two-stage methods were termed two-stage ELM (T-ELM), two-stage MARS (T-MARS), two-stage XGBoost (T-XGBoost), two-stage SGB (T-SGB), and two-stage KNN (T-KNN).

Next, we compare the performance of the models after obtaining prediction results from the five single and two-stage models under a specific game-lag. This study used the mean absolute percentage error (MAPE) as the indicator to evaluate the performance of the prediction models and determine the best game-lag selection.

(5) $$\mathrm{MAPE}\;=\frac{1}{m}\;\overset{m}{\underset{i=1}{\sum }}\left(\left|\frac{{\overset{´}{y}}_{i}-y_{i}}{y_{i}}\right|\right)\times 100$$

where ${\overset{´}{y}}_{i}$ represents the actual game score of the *i*-th sample, $y_{i}$ represents the predicted game score of the *i*-th sample, and *m* is the number of samples.

MAPE has been widely used as a performance indicator for evaluating forecasting/predicting methods [68]. When MAPE < 10%, a model is considered to have “high accurate prediction ability”. When 11% < MAPE < 20%, a model has a “good prediction ability”. When 21% < MAPE < 50%, a model has a “reasonable prediction ability”. When MAPE > 51%, a model has an “inaccurate prediction ability” [69]. A 10-fold cross-validation method is used in this study to evaluate the performances of the 10 proposed models.

In the final step of the proposed scheme, after comparing the prediction performances of the 10 models, including S-ELM, S-MARS, S-XGBoost, S-SGB, S-KNN, T-ELM, T-MARS, T-XGBoost, T-SGB and T-KNN, under different game-lags, the model with the best prediction performance is identified. Moreover, the most suitable basketball game-lag information and corresponding important basketball features are selected.

## 5. Empirical Results

In this paper, the NBA teams’ statistics in each game in the 2018–2019 season were used to verify the proposed basketball prediction scheme for predicting the final score of the NBA games. The performance of the 10 models, including S-ELM, S-MARS, S-XGBoost, S-SGB, S-KNN, T-ELM, T-MARS, T-XGBoost, T-SGB and T-KNN, can be evaluated using the proposed scheme mentioned in Section 4.

Table 3 shows the performance of the five single models, including S-ELM, S-MARS, S-XGBoost, S-SGB and S-KNN, under 1–6 game-lags. S-XGBoost obtains the best performance under game-lag = 4 with an MAPE value of 0.0842, followed by S-SGB under game-lag = 4 with an MAPE value of 0.0845, and S-MARS under game-lag = 4 with an MAPE value 0.0846.

As aforementioned, six features were selected as the important features and served as the crucial predictor variables for developing the two-stage models, including T-ELM, T-MARS, T-XGBoost, T-SGB and T-KNN. Table 4 shows the performance of the five two-stage models under six game-lags. T-XGBoost obtains the best performance under game-lag = 4 with an MAPE value of 0.0818, followed by T-SGB under game-lag = 4 with an MAPE value of 0.0829, and T-MARS under game-lag = 4 with an MAPE value of 0.0845.

From Table 3 and Table 4 it can be observed that T-XGBoost under four game-lags obtains the best performance among the models, including the single and two-stage models, under every game-lag information. It can also be seen that the 10 models obtain their lowest MAPE values, along with their best prediction performance, under game-lag = 4. Therefore, game-lag = 4 is the most suitable game-lag for NBA game score prediction.

Since the two-stage model T-XGBoost, with game-lag = 4, is the best model in this study, the six selected designed features, including ${\bar{V}}_{8,t}^{4}\;$ (defensive rebounds), ${\bar{V}}_{2,t}^{4}$ (two-point field goal percentage), ${\bar{V}}_{6,t}^{4}$ (free throw percentage), ${\bar{V}}_{7,t}^{4}$ (offensive rebounds), ${\bar{V}}_{9,t}^{4}$ (assists) and ${\bar{V}}_{3,t}^{4}$ (three-point field goal attempts), are the important features.

**Figure 3:**
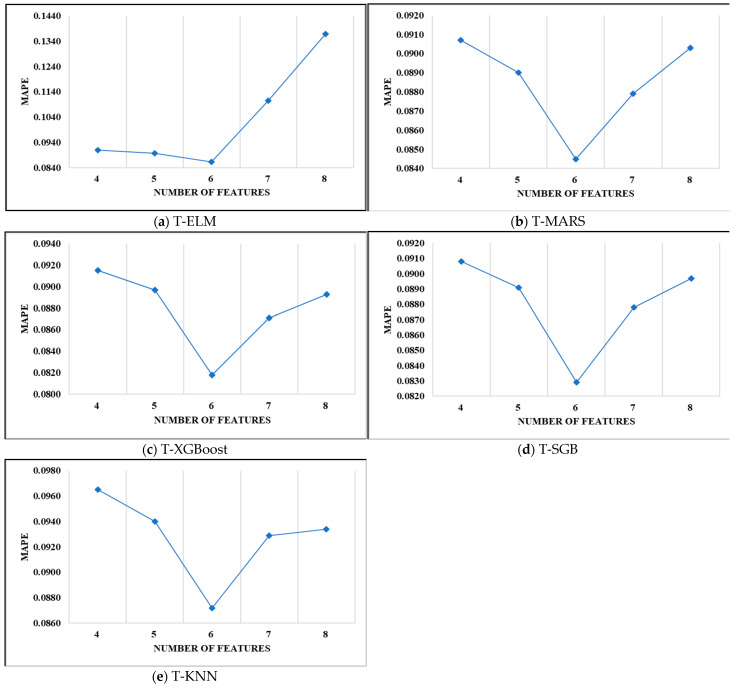
Evaluation results of the selection of different numbers of important features for modeling the two-stage models: (**a**) T-ELM, (**b**) T-MARS, (**c**) T-XGBoost, (**d**) T-SGB, (**e**) T-KNN.

To evaluate the robustness of the feature selection results of the proposed scheme, we select different numbers of important features for modeling the two-stage models according to the feature ranking results mentioned in Section 4. We consider the selection of more or fewer features than the six selected features. We used 4, 5, 6, 7 and 8 features to develop the five two-stage prediction models. Figure 3 demonstrates the MAPE value obtained by each model with a different selection of features. It shows that the MAPE value gradually decreases with an increase in the number of features selected from 4–6. The converse happens if more than six features are selected. Therefore, the selection of six features as relevant features produced better performance than any other number of features selected.

Based on the finding discussed in this manuscript, it can be inferred that the proposed basketball game outcomes prediction scheme is a promising method for the final score of NBA games. This study examines the impact of different game-lag information while related studies arbitrarily select game-lag information either by 3 or 6 [8,9]. This research suggests that game-lag = 4 is a proper selection by appropriate feature designation. The suitable important features have been selected by using feature ensemble [66] and selection techniques [67] in the proposed scheme which are simple and effective methods. These selected important features are related to offensive factors since this study is focused on predicting the final score of basketball games and this finding is supported by [13].

To further validate the effectiveness of the best model, i.e., T-XGBoost with lag = 4, of the proposed basketball game score prediction scheme, linear regression, M5P regression tree and support vector regression (SVR), which are used in related research [13], are implemented and compared.

With implementation of these three methods in the proposed scheme in Figure 1, each method will generate its own single-stage and two-stage models. The prediction performance among the S-Linear (single-stage linear regression), S-M5P (single-stage regression tree), S-SVR (single-stage SVR), T-Linear (two-stage linear regression), T-M5P (two-stage regression tree), T-SVR (two-stage SVR) and the best model T-XGBoost models under lag = 4 are compared. Alongside MAPE, we also involve root-mean-square error (RMSE) and sum of squared error (SSE) as additional performance indicators since these indicators are effectively used in sports outcomes prediction [11,13]. This study uses MAPE, RMSE and SSE as performance indicators to compare prediction performance among seven models.

The results of model comparison are shown in Table 5. As shown in Table 5, the MAPE, RMSE and SSE values of T-XGBoost are 0.0818, 11.4753 and 61,627.37, respectively. T-XGBoost still has the best prediction performance among the six competing models.

In order to test whether selecting game-lags information of four is significantly superior to other game-lag, the Wilcoxon signed-rank test is applied. The Wilcoxon signed-rank test is a distribution-free, non-parametric technique which determines whether two models are different by comparing the signs and ranks of prediction values. The Wilcoxon signed-rank test is one of the most popular tests in evaluating the predictive capabilities of two different models [70]. 

We employ the test to evaluate the prediction performance of selecting game-lag as four with other game-lag information on T-XGBoost. Table 6 shows the Z statistic and *p*-values of the two-tailed Wilcoxon signed-rank test for MAPE values between the game-lag = 4 and other game-lags. It can be observed from Table 6 that the MAPE values of the game-lag = 4 is significantly different from other game-lag. Therefore, we can conclude that selecting game-lag = 4 is significantly better than other selections of game-lag information.

To further validate the superiority of the best model, i.e., T-XGBoost with lag = 4, we test the best model with T-Linear, T-MARS, T-SVR, T-SGB, T-KNN, T-ELM and T-M5P with lag = 4 using the Wilcoxon signed-rank test. Table 7 reports the test results between the best model to each of the seven competing models. It shows that the prediction error of T-XGBoost model is significantly lower than those of its competing models. Therefore, it can be concluded that the proposed T-XGBoost model significantly outperforms the alternatives in NBA game score prediction.

## 6. Discussion and Conclusions

This research proposed a hybrid data-mining-based scheme for predicting the final score of an NBA game. We design features from original basketball statistics based on game-lag information. The proposed prediction scheme used five data mining algorithms, namely, ELM, MARS, XGBoost, SGB and KNN. The prediction scheme comprises five single and five two-stage prediction models. Empirical results showed that the T-XGBoost model using game-lag = 4 achieved the best prediction performance among the 10 competing models, using 2–6 game-lags’ information. The most suitable count of game-lag information for NBA game score prediction is four. The six identified important statistics (features) based on four game-lags are averaged defensive rebounds, averaged two-point field goal percentage, averaged free throw percentage, averaged offensive rebounds, averaged assists, and averaged three-point field goal attempts. The findings of this study may be applied to the development of several applications for other teams or even individual sports.

Since the NBA data adopted in this study are limited to one season, future research should investigate the performance of the proposed basketball game score prediction scheme with more NBA seasons. Moreover, using more seasons’ data to generate more stable important feature selection results could also be a future research direction. Furthermore, this study collected individual NBA team data to predict individual teams’ final score in NBA games. To further predict interesting or specific games’ scores or win/loss, such as particular matchups, low-scoring games, or intensity of a team’s game schedule and use these specific types of dataset to improve the performance of the proposed model, as well as making modification and extension of the proposed scheme such as considering opponent teams’ information as features, could be one of future research directions.

## Figures and Tables

**Table 1:** Variables description.

Variables	Definition	Description
$V_{1,t}$	2PA	2-Point Field Goal Attempts of a team in *t*-th game
$V_{2,t}$	2P%	2-Point Field Goal Percentage of a team in *t*-th game
$V_{3,t}$	3PA	3-Point Field Goal Attempts of a team in *t*-th game
$V_{4,t}$	3P%	3-Point Field Goal Percentage of a team in *t*-th game
$V_{5,t}$	FTA	Free Throw Attempts of a team in *t*-th game
$V_{6,t}$	FT%	Free Throw Percentage of a team in *t*-th game
$V_{7,t}$	ORB	Offensive Rebounds of a team in *t*-th game
$V_{8,t}$	DRB	Defensive Rebounds of a team in *t*-th game
$V_{9,t}$	AST	Assists of a team in *t*-th game
$V_{10,t}$	STL	Steals of a team in *t*-th game
$V_{11,t}$	BLK	Blocks of a team in *t*-th game
$V_{12,t}$	TOV	Turnovers of a team in *t*-th game
$V_{13,t}$	PF	Personal Fouls of a team in *t*-th game
$Y_{t}$	Score	Team Score of a team in *t*-th game

**Table 2:** Feature rank by MARS, XGBoost and SGB methods under game-lag = 4.

Designed Feature	MARS	XGBoost	SGB	Average Rank
${\bar{V}}_{1,t}^{4}$	6	8	10	8.00
${\bar{V}}_{2,t}^{4}$	2	1	3	2.00
${\bar{V}}_{3,t}^{4}$	5	6	8	6.33
${\bar{V}}_{4,t}^{4}$	8	7	5	6.67
${\bar{V}}_{5,t}^{4}$	7	9	7	7.67
${\bar{V}}_{6,t}^{4}$	3	4	4	3.67
${\bar{V}}_{7,t}^{4}$	4	5	6	5.00
${\bar{V}}_{8,t}^{4}$	1	2	2	1.67
${\bar{V}}_{9,t}^{4}$	13	3	1	5.67
${\bar{V}}_{10,t}^{4}$	13	13	13	13.00
${\bar{V}}_{11,t}^{4}$	13	10	11	11.33
${\bar{V}}_{12,t}^{4}$	13	11	12	12.00
${\bar{V}}_{13,t}^{4}$	9	12	9	10.00

**Table 3:** Performance of the five single models under six game-lags.

Methods	l=1	l=2	l=3	l=4	l=5	l=6
S-ELM	0.1020	0.0960	0.0915	0.0870	0.0931	0.0928
S-MARS	0.0910	0.0909	0.0897	0.0846	0.0917	0.0907
S-XGBoost	0.0919	0.0907	0.0911	**0.0842**	0.0927	0.0920
S-SGB	0.0910	0.0925	0.0913	0.0845	0.0923	0.0908
S-KNN	0.0992	0.1011	0.0947	0.0873	0.0934	0.0941

Note: The bold indicates the best prediction performance.

**Table 4:** Performance of the five two-stage models under six game-lags.

Methods	l=1	l=2	l=3	l=4	l=5	l=6
T-ELM	0.1206	0.0924	0.0951	0.0863	0.0972	0.0902
T-MARS	0.0917	0.0911	0.0912	0.0845	0.0928	0.0900
T-XGBoost	0.0918	0.0930	0.0916	**0.0818**	0.0929	0.0920
T-SGB	0.0909	0.0918	0.0912	0.0829	0.0930	0.0908
T-KNN	0.0998	0.0984	0.0973	0.0872	0.0993	0.0970

Note: The bold indicates the best prediction performance.

**Table 5:** Comparison of prediction performance of T-XGBoost and the six competing models.

Models (Lag = 4)	MAPE	RMSE	SSE
S-Linear	0.0897	12.7324	75,868.89
T-Linear	0.0883	12.0904	68,410.85
S-M5P	0.0922	13.0613	79,839.95
T-M5P	0.0931	12.9102	78,,003.64
S-SVR	0.0914	13.0213	79,351.58
T-SVR	0.0889	12.2547	70,283.06
T-XGBboost	0.0818	11.4753	61,627.37

**Table 6:** Wilcoxon singed-rank test between six pieces of game-lag information on the T-XGBoost model.

T-XGBboost	Lag = 1	Lag = 2	Lag = 3	Lag = 5	Lag = 6
Lag = 4	−1.017 (0.007) **	−1.044 (0.008) **	−4.284 (0.001) **	−6.115 (0.001) **	−10.859 (0.001) **

Note: The numbers in parentheses are the corresponding *p*-value: ** *p* < 0.05.

**Table 7:** Wilcoxon sing-rank test between T-XGBoost, T-Linear, T-MARS, T-SVR, T-SGB, T-KNN, T-ELM and T-M5P models.

Lag = 4	T-Linear	T-MARS	T-SVR	T-SGB	T-KNN	T-ELM	T-M5P
T-XGBboost	−1.239 (0.001) **	−0.994 (0.010) **	−0.997 (0.010) **	−0.989 (0.011) **	−0.885 (0.021) **	−1.377 (0.000) **	−1.043 (0.008) **

Note: The numbers in parentheses are the corresponding *p*-value; **: *p* < 0.05.

## Data Availability

Publicly available datasets were analyzed in this study. This data can be found here: https://www.basketball-reference.com, accessed on 15 March 2021.
